# Brain Functional Connectivity in Middle-Aged Hong Chuan Tai Chi Players in Resting State

**DOI:** 10.3390/ijerph191912232

**Published:** 2022-09-27

**Authors:** Weiqi Chen, Xianliang Zhang, Hui Xie, Qiang He, Zhenguo Shi

**Affiliations:** 1 School of Physical Education, Shandong University, Jinan 250062, China; 2 Beijing Key Laboratory of Rehabilitation Technical Aids for Old-Age Disability, National Research Center for Rehabilitation Technical Aids, Beijing 100176, China

**Keywords:** Tai Chi, brain functional connectivity, functional near-infrared spectroscopy, wavelet phase coherence

## Abstract

Tai Chi is an effective strategy for slowing cognitive decline, although the underlying mechanism remains unclear. We designed a cross-sectional study to examine brain functional connectivity in middle-aged Hong Chuan Tai Chi practitioners. Eighteen middle-aged Hong Chuan Tai Chi practitioners and 22 age-matched Tai Chi-naïve controls completed functional near-infrared spectroscopy (fNIRS) tests to evaluate oxyhemoglobin changes in the prefrontal cortex (PFC), motor cortex (MC), and occipital cortex (OC) in five frequency intervals (I, 0.6–2 Hz; II, 0.145–0.6 Hz; III, 0.052–0.145 Hz; IV, 0.021–0.052 Hz; V, 0.0095–0.021 Hz). Wavelet phase coherence was used to analyze the match between the instantaneous phases of the two signals to accurately measure brain functional connectivity. Global cognition was measured using the Montreal Cognitive Assessment scale. Compared with the control group, Hong Chuan Tai Chi practitioners had better global cognition (*p* < 0.01) and showed higher functional connectivity of the PFC, MC, and OC in intervals I, III, VI, and V in the resting state within the same brain hemispheres or between the left and right hemispheres. Our findings revealed that middle-aged Hong Chuan Tai Chi practitioners had higher functional connectivity of the PFC, MC, and OC across both brain hemispheres in cardiac activity, myogenic activity, sympathetic nervous system, and endothelial cell metabolic activities which may contribute to higher global cognition.

## 1. Introduction

Cognition is one of the most important health variables, and age-related cognitive decline has become a major public health concern worldwide. In China, the overall prevalence of mild cognitive impairment and dementia was estimated to be 15.5% (38.77 million people) and 6% (15.07 million people), respectively [1]. Brain functions often decline with aging, which may lead to cognitive impairments and even dementia [2]. The degradation of brain function reduces the quality of life of older adults. The lack of effective pharmaceutical treatments to alleviate or stop brain function degradation and cognitive decline has contributed to a growing interest in low-cost behavioral interventions.

Tai Chi, also known as Tai Chi Chuan or Taijiquan, developed as an ancient Chinese martial art and widely practiced today for its health benefits. Tai Chi incorporates physical, cognitive, social, and meditative components as a multimodal mind-body exercise. Practicing Tai Chi has multiple benefits on cognitive function in both young and older adults [3,4]. Tai Chi provides aerobic and agility training and involves learning choreographed movement patterns that support visuospatial processing, processing speed, and episodic memory. In addition, practicing Tai Chi requires sustained attentional control to complete multiple tasks. Meditation can help improve attention and executive function by increasing the brain’s ability to allocate attentional resources. Moreover, Tai Chi practice has been observed to affect the prefrontal structure and function and improve memory by functional magnetic resonance imaging(fMRI) [5,6]. In addition, Tai Chi practitioners had thickened parietal and occipital cortices [6]. However, the underlying mechanisms remain poorly understood. The formation of functional brain networks is closely associated with cognitive behavior [7]. Examining neural connectivity patterns provides valuable insights into the benefits of Tai Chi on brain function and cognitive function.

Functional near-infrared spectroscopy (fNIRS) is a non-invasive brain function imaging technology that can continuously monitor brain activity at rest or during tasks. It uses specific wavelengths of light to measure cerebral oxyhemoglobin (HbO_2_) and deoxyhemoglobin. HbO_2_ changes (Δ[HbO_2_]) across several brain regions and their temporal correlation can be easily obtained and analyzed to evaluate brain functional connectivity. The resting-state reflects spontaneous brain activity and is a vital experimental paradigm in the study of brain function. The fNIRS signals obtained from cortical regions during the resting state mainly reflect regional hemodynamic fluctuations originating from spontaneous neural activity [8]. There are strong correlations among the hemoglobin oxygenation signal fluctuations of distinct brain regions at low frequencies in the resting state. This correlation, termed “resting-state functional connectivity,” has been reproduced in many studies using fNIRS [9,10]. Spontaneous fluctuations were often found in the spectral analysis of Δ[HbO_2_] signals in the resting brain [11,12]. The power spectra of Δ[HbO_2_] signals exhibited fluctuations in various frequency bands, indicating possible regulatory mechanisms of cerebral oxygenation signals. For example, the oscillations in intervals I (0.6–2 Hz) and II (0.145–0.6 Hz) reflected the synchronization of cardiac and respiratory activities, respectively [11].The oscillations in interval III (0.05–0.15 Hz) originate locally from the intrinsic myogenic activity of smooth muscle cells in resistance vessels. Interval IV (0.02–0.05 Hz) was closely regulated through tight neurovascular coupling and partial autonomic control, whereas Interval V (0.0095–0.021 Hz) was regarded as endothelial cell metabolic activity. Thus, spontaneous oscillations in cerebral hemodynamic signals may be used to monitor brain functional activation. The quantification of fNIRS information can be estimated using a wavelet transform. Wavelet coherence (WCO) reveals the strength of two signals at the same frequency. In contrast, wavelet phase coherence (WPCO) localizes the phase-locked behavior, revealing possible relationships by evaluating the match between the instantaneous phases of the two signals. WPCO analysis has been used to analyze the relationship between oscillations in brain oxygen saturation within certain specific frequency ranges [13,14].

The regions of interest (ROIs) were the prefrontal cortex (PFC), motor cortex (MC), and occipital cortex (OC), which are closely involved in human physical behavior activities. The PFC is the central cognitive region and plays an important role in decision-making, cognitive control, and linking cognition to action. Its cortico-cortical connections reach the primary MC and transfer crucial information to execute motor output [15]. The MC is located on both sides of the brain’s central sulcus and is critical for planning and executing voluntary movements. However, movement planning is supported by multiple regions [16]. The OC is related to visual perception in motion, and an occipitomotor connection can be activated during exercise [17] or transcranial magnetic stimulation [18]. The connectivity between these functional cortices is critical for movement coordination, which Tai Chi requires. A previous study reported that the functional connectivity between these three regions in Tai Chi practitioners was higher than in Tai Chi-naïve controls in adults aged 60–70 years [17]. However, there are several forms of Tai Chi, such as Chen style, Yang style, Wu style, Sun style, etc. These different types of Tai Chi have their own different characteristics, whereas deep diaphragmatic breathing, relaxation, and the imperceptibly smooth flow of body postures are common signature features. Most existing studies have examined Chen styles and Yang styles of Tai Chi, and consistently reported benefits to brain function [19,20,21]. Different forms of exercise induce different modulations of brain function involved in cognitive control, such as Tai Chi and Baduanjin [22], and modified Chen-style and 24-style Tai Chi [21]. Hong Chuan Tai Chi originated from Chen-style Tai Chi and is quite popular in Jinan, Shandong province, China. As a branch of Chen-style Tai Chi, this 81-form Tai Chi emphasizes the art of attachment and defense and is much more strenuous with a longer practice time than other forms of Tai Chi. Thus, the effects of Hong Chuan Tai Chi practice on brain function in the 50–60 year old middle-aged population are worth exploring. However, no studies have explored the health benefits of Hong Chuan Tai Chi. Our hypothesis is that Hong Chuan Tai Chi practitioners will have better brain functional connectivity than their aged-matched Tai Chi-naïve controls.

## 2. Materials and Methods

### 2.1. Participants and Design

This study was a part of a cooperative research program run in 2017, and all participants were recruited from a community in Jinan city, Shandong Province, China. The sample size was calculated by G.power and required 29 subjects per group. However, some participants dropped out due to personal reasons. The inclusion criteria were as follows: (i) middle-aged adults aged 50 to 60 years old; (ii) no hypertension, history of brain trauma and nervous system diseases, history of neurological drugs or sleeping pills in the past three years, and drug abuse; (iii) Tai Chi Chun (TCC) practitioners needed at least three years of experience in Hong Chuan Tai Chi, whereas control (C) participants needed to have no history of regular physical activity, yoga, or mediation treatment; (iv) all participants needed to be right-handed. Previous studies have suggested that right-handed and left-handed people have different brain functioning between their right and left hemispheres. It is well-established that the functioning of the left hemisphere is reversed in left-handed people. Therefore, to control the possible variability, we excluded participants with left-handedness. A total of 40 healthy middle-aged adults (50–60 years old) completed this fNIRS study, including 18 Hong Chuan Tai Chi practitioners (55.78 ± 2.64 years old) and 22 demographically matched Tai Chi-naïve healthy controls (54.69 ± 3.10 years old). On average, the Tai Chi practitioners had 4.61 years (SD = 0.89) of experience with Hong Chuan Tai Chi practice (Table 1).

All the participants were informed of the specific contents of the experiment in advance and provided written informed consent. The experiment was conducted in accordance with the Declaration of Helsinki and was approved by the ethics committee of the Rehabilitation Hospital affiliated with the National Research Center for Rehabilitation Technical Aids. The ethics committee approved all experimental procedures in this study.

Basic information, including sex, age, body weight, height, educational background, and medical history, was self-reported. Sleep quality was evaluated using a Chinese version of the Pittsburgh Sleep Quality Index (PSQI), which has already been validated and is widely used in the Chinese population [23]. Global cognitive status was assessed using the Montreal Cognitive Assessment (MoCA) scale. Both PSQI and MoCA were performed by a senior psychiatrist. Resting blood pressure was measured using an electronic sphygmomanometer. Each participant was familiarized with the experimental protocol. All subjects who met the inclusion criteria had a MoCA score of 26 or above, and PSQI scores ranged from 1 to 6. Before starting the fNIRS test, all participants were required to maintain a comfortable sitting position, close their eyes, and remain awake. After the instrument was placed, all participants were required to remain sitting to collect 15 min of resting-state data (without systematic thinking, in a relaxed state). Continuous recordings of fNIRS signals were then obtained from the left and right PFC, MC, and OC of the 18 Hong Chuan Tai Chi practitioners and 22 age-matched controls. The same technician performed the fNIRS measurements.

### 2.2. Procedure and Measurements

Before performing the fNIRS test, all participants completed a sleep quality survey. The PSQI is a self-rated questionnaire used to evaluate the sleep quality of subjects over a 1-month time interval and has been validated as a reliable test for identifying sleep disorders in clinical and research settings. Nineteen individual items generate seven components with a score range of 0–21. A higher score indicates worse sleep quality, with scores lower than six indicating good sleep quality [24].

### 2.3. Functional Near-Infrared Spectroscopy

**Figure 1:**
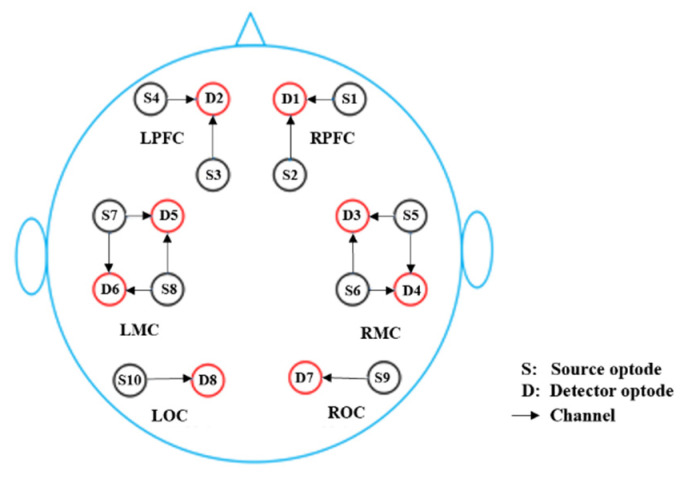
Probe arrangement of multichannel blood oxygen detection system. The red circle represents the detector, the black circle represents the light source, and the arrow is the channel of the light source pointing to the detector.

A multi-channel continuous-wave tissue oxygenation monitor (NirSmart, Danyang Huichuang Medical Equipment Co., Ltd., Danyang, China) was used for fNIRS measurements. The details are elaborated in our previous study [17]. Each instrument sensor consisted of a three-wavelength light-emitting diode, which emitted light at 740, 808, and 850 nm wavelengths, and a detector optode. The 14-channels of the fNIRS were symmetrically positioned over the left PFC (LPFC), MC (LMC), and OC (LOC), and the right PFC (RPFC), MC (RMC), and OC (ROC). All protocols were performed by a well-trained technician, following the guidelines for clinical laboratory safety. Figure 1 shows the distribution of the 14 channels in the brain, consisting of 10 light source probes and 8 detector probes. The sampling frequency was 10 Hz.

### 2.4. Data Preprocessing

The fNIRS signal was mixed with system physiological and tissue noise because some physiological activity cannot be stopped. This inevitably affects the hemodynamics, especially in the task state. For example, cardiac activity (0.8~2 Hz) and respiration (0.13~0.33 Hz) can induce global drift in the baseline measurements. In addition, before the near-infrared light reaches grey matter, it needs to pass through the scalp, subcutaneous tissue, skull, and cerebrospinal fluid, influencing the fNIRS signal. Finally, spontaneous neural activity in the brain might mix cognitive task-induced neuroactivity, such as low-frequency oscillations (~0.1 Hz) and very-low-frequency oscillations (~0.03 Hz), although they can be used in the resting state. Data pre-processing aimed to remove signals with a low signal-noise ratio to accurately evaluate brain activity-induced hemodynamic responses. The data were preprocessed using the moving average method for subsequent analyses to remove these effects, as described in previous studies [25]. The time window used for the moving average filter was 3 s. A processing method based on the moving standard deviation and cubic spline interpolation was applied to remove motion artifacts. The artifact portion was determined by identifying the standard deviation of the sliding window above a certain threshold and was removed by cubic spline interpolation. Then, independent component analysis was performed on the Δ[HbO_2_] signals of each channel to reduce physiological interference in fNIRS measurements. A sixth-order Butterworth band-pass filter was used to obtain filtered signals of 0.005–2 Hz with an improved signal-to-noise ratio and remove elements (below 0.005 Hz and above 2 Hz) of the raw signal.

### 2.5. Functional Connectivity Analysis

The functional connectivity was calculated using a wavelet-based coherence method, as described in previous studies [13]. After data preprocessing, wavelet transformation (WT) was used to transform the preprocessed data in a time series, from the time domain to the time-frequency domain. Then, the fNIRS signals from the left and right PFC, MC, and OC were analyzed according to five frequency intervals: I, 0.6–2 Hz; II, 0.145–0.6 Hz; III, 0.052–0.145 Hz; IV, 0.021–0.052 Hz; V, 0.0095–0.021 Hz. The physiological meanings of each frequency interval were cardiac activity, respiration, vascular smooth muscle activity, neural activity, and endothelial cell metabolic activity. WPCO was used to detect the degree of correlation between two signals at a certain frequency interval, which represents the instantaneous phase of the two signals at a consistent degree through the continuous process of the time series to identify possible connectivity. The value of WPCO is between 0 and 1 and reflects the instantaneous difference between the two Δ[HbO_2_] signals as a constant trend through the study. A higher WPCO value indicates that a stronger functional connectivity between the two brain regions exists; otherwise, it indicates that less of a relationship exists between the two Δ[HbO_2_] signals.

### 2.6. Statistical Analysis

All data were analyzed using SPSS20.0 software (SPSS Inc., Chicago, IL, USA). Shapiro–Wilk tests were used for a normality of distribution test; when the main analysis variables did not show a normal distribution, a nonparametric analysis was performed. Otherwise, data was analyzed using an independent t-test. The results are expressed as mean ± SD. Statistical significance was set at *p* < 0.05. Cohen’s d and effect sizes were calculated for each dependent variable. Pearson’s correlation analysis was performed to examine the relationship between cognitive function and functional connectivity in different brain regions.

## 3. Results

The demographic characteristics of all participants in each group are analyzed and summarized in Table 1. A total of 40 participants completed the fNIRS study. All participants were instructed to maintain their dietary habits and physical activity, if any, throughout the study. There were no significant differences in age, years of education, body mass index (BMI), resting blood pressure, or sleep quality between the two groups.

Participant MoCA performance is presented in Table 2. Hong Chuan Tai Chi practitioners scored higher on the MoCA test (*p* < 0.001) than healthy Tai Chi-naïve controls, with a range of 26–30 in their total score compared with 25–28. They showed significantly better performance in the test subdomains of visual spatial (*p* < 0.01) and memory (*p* < 0.05). No significant difference was observed in terms of naming, attention, language, abstraction, and orientation between the Hong Chuan Tai Chi practitioners and healthy Tai Chi-naïve controls.

### Wavelet Phase Coherence Analysis

**Figure 2:**
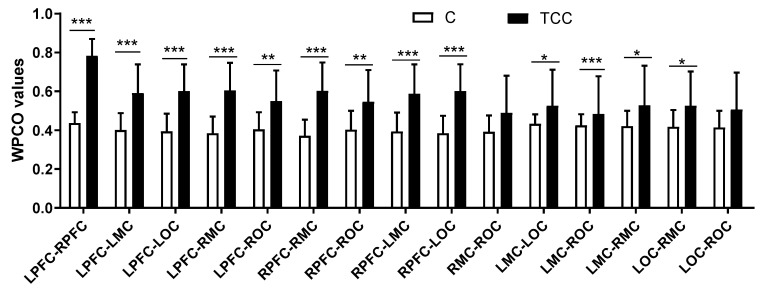
Comparison of the WPCO values between different brain regions at frequency interval I in resting state. * (*p* < 0.05), ** (*p* < 0.01), and *** (*p* < 0.001) indicate significant differences between the Tai Chi and control groups.

As Figure 2 shows, in interval I, Hong Chuan Tai Chi practitioners had significantly higher WPCO values of the LPFC–LMC (TC = 0.59, C = 0.40, *p* < 0.001, Cohen’s d = 1.54, effect size = 0.61), LPFC–LOC (TC = 0.60, C = 0.39, *p* < 0.001, Cohen’s d = 1.78, effect size = 0.61), and LMC–LOC (TC = 0.53, C = 0.43, *p* < 0.05, Cohen’s d = 0.76, effect size = 0.35) in the left hemisphere, compared with Tai Chi-naïve controls.

Hong Chuan Tai Chi practitioners also had significantly higher WPCO values of the RPFC–RMC (TC = 0.60, C = 0.37, *p* < 0.001, Cohen’s d = 1.91, effect size = 0.69) and RPFC–ROC (TC = 0.55, C = 0.40, *p*< 0.01, Cohen’s d = 1.12, effect size = 0.49) in the right hemisphere.

In addition, Hong Chuan Tai Chi practitioners had significantly higher WPCO values of the LPFC–RPFC (TC = 0.78, C = 0.44, *p* < 0.001, Cohen’s d = 4.44, effect size = 0.91), LPFC–RMC (TC = 0.60, C = 0.38, *p* < 0.001, Cohen’s d = 1.87, effect size = 0.68), LPFC–ROC (TC = 0.55, C = 0.40, *p* < 0.01, Cohen’s d = 1.16, effect size = 0.50), RPFC–LMC(TC = 0.59, C = 0.39, *p* < 0.001, Cohen’s d = 1.57, effect size = 0.62), RPFC–LOC (TC = 0.60, C = 0.38, *p* < 0.001, Cohen’s d = 1.87, effect size = 0.68), LMC–RMC (TC = 0.53, C = 0.42, *p* < 0.05, Cohen’s d = 0.77, effect size = 0.36), and LOC–RMC (TC = 0.53, C = 0.42, *p* < 0.05, Cohen’s d = 0.77, effect size = 0.36) between the two hemispheres compared with Tai Chi-naïve controls.

**Figure 3:**
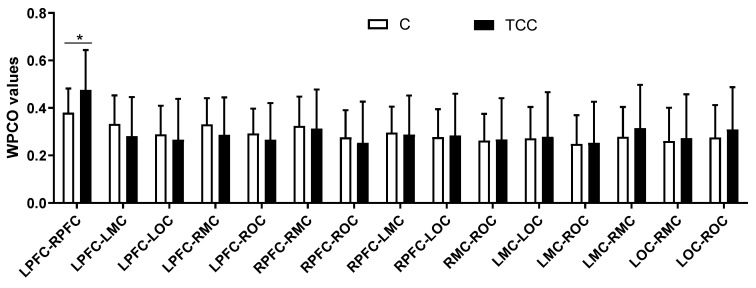
Comparison of the WPCO values between different brain regions at frequency interval II in resting state. * (*p* < 0.05) indicate significant differences between the Tai Chi and control groups.

As shown in Figure 3, Hong Chuan Tai Chi practitioners had significantly higher WPCO values of the LPFC–RPFC (TC = 0.48, C = 0.38, *p* < 0.05, Cohen’s d = 0.72, effect size = 0.34) at interval II, compared with Tai Chi-naïve controls. There were no significant differences between other brain regions in interval II.

**Figure 4:**
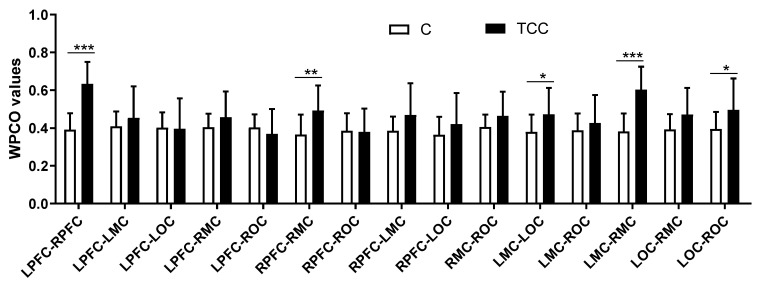
Comparison of the WPCO values between different brain regions at frequency interval III in resting state. * (*p* < 0.05), ** (*p* < 0.01), and *** (*p* < 0.001) indicate significant differences between the Tai Chi and control groups.

Figure 4 shows that in interval III, Hong Chuan Tai Chi practitioners had significantly higher WPCO values of the LMC–LOC (TC = 0.47, C = 0.38, *p* < 0.05, Cohen’s d = 0.76, effect size = 0.36) and RPFC–RMC (TC = 0.49, C = 0.37, *p* < 0.01, Cohen’s d = 1.00, effect size = 0.45) than in Tai Chi-naïve controls in the left and right hemisphere, respectively. In addition, Hong Chuan Tai Chi practitioners had significantly higher WPCO values of the LPFC–RPFC (TC = 0.63, C = 0.39, *p* < 0.001, Cohen’s d = 2.26, effect size = 0.75), LMC–RMC (TC = 0.60, C = 0.38, *p* < 0.001, Cohen’s d = 1.99, effect size = 0.71), LOC–RMC (TC = 0.47, C = 0.39, *p* < 0.05, Cohen’s d = 0.70, effect size = 0.33), and LOC–ROC (TC = 0.50, C = 0.40, *p* < 0.05, Cohen’s d = 0.74, effect size = 0.35) between the two hemispheres than in Tai Chi-naïve controls.

**Figure 5:**
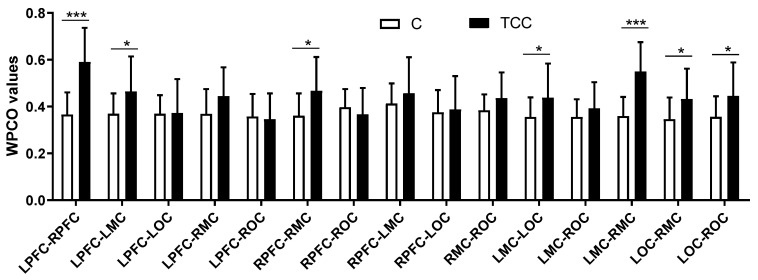
Comparison of the WPCO values between different brain regions at frequency interval IV in resting state. * (*p* < 0.05) and *** (*p* < 0.001) indicate significant differences between the Tai Chi and control groups.

Figure 5 shows the comparison of WPCO values between the Hong Chuan Tai Chi and control groups at interval IV. Compared with Tai Chi-naïve controls, Hong Chuan Tai Chi practitioners had significantly higher WPCO values of the LPFC–LMC (TC = 0.46, C = 0.37, *p* < 0.05, Cohen’s d = 0.73, effect size = 0.34), LMC–LOC (TC = 0.44, C = 0.36, *p* < 0.05, Cohen’s d = 0.67, effect size = 0.32), and RPFC–RMC (TC = 0.47, C = 0.36, *p* < 0.05, Cohen’s d = 0.90, effect size = 0.41) in the left and right hemisphere, respectively. In addition, Hong Chuan Tai Chi practitioners had significantly higher WPCO values of the LPFC–RPFC (TC = 0.59, C = 0.37, *p* < 0.001, Cohen’s d = 1.78, effect size = 0.66), LMC–RMC (TC = 0.55, C = 0.36, *p* < 0.001, Cohen’s d = 1.76, effect size = 0.66), LOC–RMC (TC = 0.43, C = 0.35, *p* < 0.05, Cohen’s d = 0.72, effect size = 0.34), and LOC–ROC (TC = 0.45, C = 0.36, *p* < 0.05, Cohen’s d = 0.76, effect size = 0.36) between the two hemispheres compared with Tai Chi-naïve controls.

**Figure 6:**
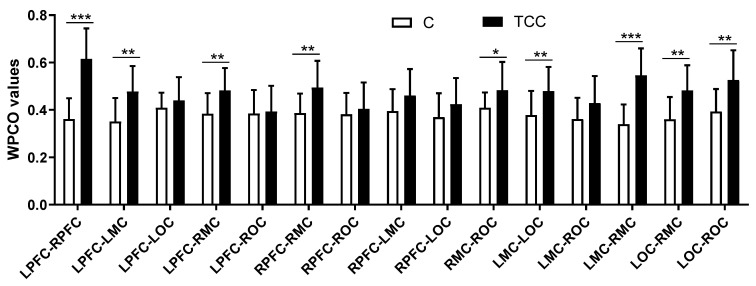
Comparison of wavelet phase coherence values between different brain regions at frequency interval V in resting state. * (*p* < 0.05), ** (*p* < 0.01), and *** (*p* < 0.001) indicate significant differences between the Tai Chi and control groups.

As shown in Figure 6, Hong Chuan Tai Chi practitioners had significantly higher WPCO values of the LPFC–LMC (TC = 0.48, C = 0.35, *p* < 0.01, Cohen’s d = 1.24, effect size = 0.53) and LMC–LOC (TC = 0.48, C = 0.38, *p* < 0.01, Cohen’s d = 1.00, effect size = 0.45) in the left hemisphere, at interval V. Additionally, they had significantly higher WPCO values of the RPFC–RMC (TC = 0.49, C = 0.39, *p* < 0.01, Cohen’s d = 1.04, effect size = 0.46) and RMC–ROC (TC = 0.48, C = 0.41, *p* < 0.05, Cohen’s d = 0.79, effect size = 0.37) in the right hemisphere. In addition, Tai chi practitioners had significantly higher WPCO values of the LPFC–RPFC (TC = 0.62, C = 0.36, *p* < 0.001, Cohen’s d = 2.33, effect size = 0.76), LPFC–RMC (TC = 0.48, C = 0.38, *p* < 0.01, Cohen’s d = 1.11, effect size = 0.49), LMC–RMC (TC = 0.55, C = 0.34, *p* < 0.001, Cohen’s d = 2.18, effect size = 0.74), LOC–RMC (TC = 0.48, C = 0.36, *p* < 0.01, Cohen’s d = 1.19, effect size = 0.51), and LOC–ROC (TC = 0.53, C = 0.39, *p* < 0.01, Cohen’s d = 1.21, effect size = 0.52) between the two hemispheres than in Tai Chi-naïve practitioners.

## 4. Discussion

We performed a cross-sectional study to examine differences in brain functional connectivity between Hong Chuan Tai Chi practitioners and age-matched Tai Chi-naïve controls in the resting state. Our results suggest that 50–60 year old Hong Chuan Tai Chi practitioners had better global cognitive abilities than age-matched Tai Chi-naïve controls, which may be associated with the higher brain functional connectivity of the PFC, MC, and OC, evidenced by the correlational analyses.

Tai Chi is a prospective strategy for delaying the decline of cognitive function in healthy adults and individuals with cognitive impairment. An early cross-sectional study compared the memory functioning of older adults aged over 56 who regularly practiced mind-body or cardiovascular exercises with those who did not engage in regular exercise. Older adults who practiced Tai Chi showed better memory functioning [26]. Another cross-sectional study suggested that a Tai Chi group demonstrated better performance on the Mini-Mental Status Examination, Color Trails Form attention test, and Rivermead Behavioral Memory Test than the other exercise or control groups [27]. Older adults with longer mind-body exercise habits usually scored higher on most cognitive tests [28]. In the current study, we screened the global cognitive abilities of 50–60 year old adults using the MoCA. The MoCA is a 30-item test that has been extensively used to assess cognitive impairments in adults in past research studies [21,29]. Consistent with previous cross-sectional and cohort studies [26,30], our study found that Hong Chuan Tai Chi practitioners had a better global cognitive status than Tai Chi-naïve controls. This study cannot claim a causal relationship; however, we suggest that there is a close association between practicing Hong Chuan Tai Chi and cognitive benefits.

Age-related changes in brain functional connectivity of older adults have been widely studied in recent years, especially using functional magnetic resonance imaging(fMRI) in resting state. Most studies have demonstrated that brain functional connectivity decreases in older adults; however, increased connectivity has been observed in some cases [31,32]. Most evidence from functional magnetic resonance imaging(fMRI) has revealed that the decreases in general brain functional connectivity may be associated with declined cognitive performance in healthy older adults [32,33]. In recent years, fNIRS studies have also suggested that brain functional connectivity decreases in older adults, but most have focused on established networks, such as the default mode network(DMN), dorsal attention network(DAN), or the salience network (SN) [34]. The functional connectivity between other brain regions have rarely been studied. In our study, we found significantly higher WPCO values of the LPFC–LMC, LPFC–LOC, LMC–LOC, RPFC–RMC, and RPFC–ROC and higher WPCO values of the LPFC–RPFC, LPFC–RMC, LPFC–ROC, RPFC–LMC, RPFC–LOC, LMC–RMC, and LOC–RMC in Hong Chuan Tai Chi practitioners, compared with matched Tai Chi-naïve controls, in interval I. As previously described, interval I oscillations reflect changes in cerebral oxygen signal responses to ab individual’s cardiac activity. As part of the cardiovascular system, the function of the cerebrovascular system in older adults declines with age, which can be altered by exercise. Our results suggest that long-term Hong Chuan Tai Chi practice may improve cardiac fitness, as reported in previous studies [35]. These training-induced benefits to the heart may include, but are not limited to, increased stroke volume. Cardiac output in the resting state contributes to a higher activation of related brain regions, and thus increases brain functional connectivity. This increased functional connectivity between the PFC, MC, and OC in interval I indicates the benefits of Hong Chuan Tai Chi on the coordination of different brain regions by modulating cardiac activity. This could be useful in illustrating positive associations between cardiovascular fitness and cognition [36].

The oscillations in interval II closely resemble the oscillations of respiratory activity. The abdominal respiration required during Hong Chuan Tai Chi can increase the intensity of diaphragm relaxation and shrinkage. This is necessary to provide adequate oxygen to move the body and increase cerebral blood flow, which induces higher cerebral Δ[HbO_2_]. A previous study suggested that short-term Tai Chi exercise can enhance pulmonary ventilation in older adults, attributed to improved respiratory muscle strength [37]. However, there was no significant difference in WPCO values between most brain regions of interest in subjects from the two groups in our study, except for LPFC–RPFC connectivity. These results suggest that the breathing activities in Tai Chi improved oxygen supply to the RPFC and LPFC. One study suggested that the breathing frequency of a skilled Tai Chi group was lower than that of the control group, but with increased breathing depth, indicating increased oxygen uptake and cerebral blood oxygen levels in the resting state [38]. Tai Chi highly values coordination of the body, mind, and breath. The “Qichen Dantian,” which means deep breathing to the belly, is an essential requirement of Tai Chi and is related to the subconscious control of the PFC [39].

The oscillations in interval III are associated with the myogenic activity of vascular smooth muscle cells [40]. The contraction and relaxation of vascular smooth muscle can influence cerebral oxygen signals by modulating the blood vessel diameter and pressure. Aging may have consequences for cerebral blood flow due to the degeneration of the vascular system. Past evidence has suggested that aging induces a reduction in very-low-frequency (0.02–0.07 Hz) and low-frequency (0.07–0.2 Hz) spontaneous oscillations of cerebral hemodynamics during cognitive tasks [41]. Decreased spontaneous activity in microvascular smooth muscle cells reflects changes in the microvasculature and increases in vessel stiffness. Long-term Tai Chi exercise may improve autonomic nervous modulation in older adults [42]. Therefore, the myogenic activity of cerebral vascular smooth muscle cells may be higher in Tai Chi practitioners than in controls. In this study, Hong Chuan Tai Chi practitioners had higher WPCO values between the LMC–LOC, RPFC–RMC, LPFC–RPFC, LMC–RMC, LOC–RMC, and LOC–ROC than in Tai Chi-naïve controls. This suggests higher functional connectivity between these regions in highly experienced Hong Chuan Tai Chi practitioners. This result is consistent with the results reported in other Tai Chi-related studies [17].

The oscillations in interval IV are related to neurogenic activity, which is regulated by neurovascular coupling and controls smooth muscle cells. These cells can release various types of vasodilator and contractile factors, which are regulated by sympathetic nerves. Robust coupling of neuronal activation to regional cerebral blood flow can be easily observed. For example, the activation of the OC by visual stimulation can elicit a 20~30% increase in blood velocity in the posterior cerebral arteries [43]. In this study, the WPCO values of the LPFC–LMC, LMC–LOC, RPFC–RMC, LPFC–RPFC, LMC–RMC, LOC–RMC, and LOC–ROC in Hong Chuan Tai Chi practitioners were significantly higher than in controls, indicating higher functional connectivity in the resting state. This suggests that long-term Hong Chuan Tai Chi practice is associated with the synchronization of neurogenic activity and PFC, MC, and OC coordination because Tai Chi requires the coordination of movement, mind, and visual perception.

The oscillations in interval V are associated with the metabolic activity of vascular endothelial cells. Endothelial cells can release the potent vasodilator nitric oxide (NO), and the NO-related metabolic activity of endothelial cells is closely associated with neurovascular activity. Previous studies have suggested that Tai Chi can improve endothelial function and arterial stiffness in elderly individuals [44]. Moreover, Hong Chuan Tai Chi improves the metabolic activity of vascular endothelial cells. In this study, Hong Chuan Tai Chi practitioners had higher WPCO values of the LPFC–LMC, LMC–LOC, RPFC–RMC, RMC–ROC, LPFC–RPFC, LPFC–RMC, LMC–RMC, LOC–RMC, and LOC–ROC than in Tai Chi-naïve controls. These results suggest higher functional connectivity between these brain regions in the Hong Chuan Tai Chi group.

However, this study had several limitations. First, although sample size was calculated in advance, some participants dropped out of the study due to personal reasons. The small sample size might have weakened the confidence of this study. In addition, a cross-sectional study design could not completely rule out confounding factors related to lifestyle and environment. A future randomized controlled trial may help rule out these confounding factors and establish a causal relationship between Hong Chuan Tai Chi practice and brain functional connectivity. In addition, as Hong Chuan Tai Chi is different from the classical Chen and Yang styles of Tai Chi, it is necessary to compare their efficiency in brain-related changes to these populations to determine the uniqueness of Hong Chuan Tai Chi.

## 5. Conclusions

This study was the first to explore the benefits of Hong Chuan Tai Chi on cognition and brain functional connectivity. This study utilized fNIRS to examine the brain functional connectivity between the PFC, MC, and OC in Hong Chuan Tai Chi practitioners and compared them with Tai Chi-naïve subjects. The study demonstrated that middle-aged Hong Chuan Tai Chi practitioners had higher functional connectivity between the PFC, MC, and OC. The stronger connectivity between the left and right brain regions may contribute to higher global cognition.

## Figures and Tables

**Table 1:** Demographic characteristics.

	TCC (*n* = 18)	C (*n* = 22)	*p*
Age (year)	55.78 ± 2.64	54.69 ± 3.10	0.401
Gender (men)	10	13	NA
Body mass (kg)	60.67 ± 6.40	61.29 ± 6.49	0.764
Height (m)	1.65 ± 0.06	1.67 ± 0.10	0.475
BMI (kg/m^2^)	22.10 ± 0.61	22.27 ± 0.68	0.556
Education (year)	12.67 ± 2.00	12.00 ± 2.12	0.467
Tai Chi experience (year)	4.61 ± 0.89	NA	NA
Duration (hour/w)	13.89 ± 1.62	NA	NA
Handedness (right)	18	22	NA
Systolic pressure (mmHg)	116.00 ± 7.38	120.85 ± 6.79	0.128
Diastolic pressure (mmHg)	73.89 ± 3.48	76.61 ± 5.38	0.197
Sleep quality (PQSI)	3.61 ± 0.78	3.82 ± 0.85	0.432

NA: Not Available.

**Table 2:** The MoCA test.

	TCC (*n* = 18)	C (*n* = 22)	*p*
Visual spatial/executive function	4.39 ± 0.61 **	3.68 ± 0.72	0.002
Naming	2.94 ± 0.24	3.00 ± 0.00	0.274
Memory	4.44 ± 0.51 *	3.91 ± 0.75	0.014
Attention	5.83 ± 0.38	5.45 ± 0.74	0.057
Language	2.67 ± 0.49	2.59 ± 0.5	0.633
Abstraction	1.89 ± 0.32	1.82 ± 0.39	0.545
Orientation	5.89 ± 0.32	5.82 ± 0.39	0.545
Total score	28.06 ± 0.64 ***	26.27 ± 1.2	0.000

* (*p* < 0.05), ** (*p* < 0.01), and *** (*p* < 0.001) indicates a significant difference between the Tai Chi and control groups.

## Data Availability

Not applicable.
